# Nordic Nutrition Recommendations and risk of myocardial infarction and stroke: a prospective cohort study

**DOI:** 10.1007/s00394-024-03337-7

**Published:** 2024-02-14

**Authors:** Linnea Sjöblom, Essi Hantikainen, Rino Bellocco, Weimin Ye, Hans-Olov Adami, Ylva Trolle Lagerros, Stephanie Erika Bonn

**Affiliations:** 1 Division of Clinical Epidemiology, Department of Medicine (Solna), Karolinska Institutet, 171 76 Stockholm, Sweden; 2 Deparment of Statistics and Quantitative Methods, University of Milano-Bicocca, 20126 Milan, Italy; 3 Institute for Biomedicine, Eurac Research, 39100 Bolzano, Italy; 4 Department of Medical Epidemiology and Biostatistics, Karolinska Institutet, 171 77 Stockholm, Sweden; 5 Clinical Effectiveness Group, Institute of Health and Society, University of Oslo, 0318 Oslo, Norway; 6 Center for Obesity, Academic Specialist Center, Stockholm Health Services, 113 65 Stockholm, Sweden

**Keywords:** Cardiovascular disease, Dietary recommendations, Epidemiology, Myocardial infarction, Nordic diet, Stroke

## Abstract

**Purpose:**

The Nordic Nutrition Recommendations (NNR) are developed to prevent diet-related diseases. This study aimed to examine adherence to the NNR and risk of myocardial infarction (MI) and stroke among women and men in Sweden.

**Methods:**

We followed 34,898 adults from 1997 to 2016. Dietary intake was assessed once at baseline using a food frequency questionnaire. Adherence scores corresponding to NNR-editions from 2023, 2012, 2004 and 1996 were calculated. Scores were categorized into low (reference category), moderate and high adherence. Cox proportional hazards regression models adjusted for potential confounders were used to estimate hazards ratios (HR) with 95% confidence intervals (CI).

**Results:**

We identified 1649 incident cases of MI and 2071 incident cases of stroke during follow-up (mean 17.9 years). For each 1-point increase in the NNR_2023_-score (range 0–9), the rate of MI decreased by 14% (HR: 0.86; 95% CI 0.78–0.95). High adherence was associated with a lower rate of MI (HR: 0.72; 95% CI 0.59–0.87) (*p*-trend = 0.01). Moderate adherence was associated with a lower rate of stroke (HR: 0.88; 95% CI 0.78–0.99) (*p*-trend = 0.31). Among women, a 23% lower rate of MI (HR: 0.77; 95% CI 0.67–0.89) was observed for each 1-point increase, and high adherence was associated with a lower rate of MI (HR: 0.59; 95% CI 0.45–0.78). No associations were found in men. The results were similar, though attenuated, for earlier NNR-editions.

**Conclusion:**

Adherence to the NNR was associated with a reduced risk of MI. This association was more pronounced among women than among men and in more recent NNR-editions. The findings for stroke need further investigation.

**Supplementary Information:**

The online version contains supplementary material available at 10.1007/s00394-024-03337-7.

## Introduction

Unhealthy diets account for about 22% of all deaths among adults worldwide [1]. Cardiovascular disease (CVD) is the leading cause of death, with more than 80% of CVD deaths caused by myocardial infarction (MI) or stroke [2]. However, MI and stroke may be prevented by a healthy diet [3]. Individual foods such as fruits, vegetables [4] or nuts [5], or single nutrients, such as dietary fiber [6] and whole grains [7], have been shown to reduce CVD risk. On the contrary, a high intake of salt has been significantly associated with an increased risk of CVD, and stroke in particular [8]. However, considering the interplay between different components of the diet, it may be more valuable to examine the effect of the overall diet rather than single nutrients [9]. The Mediterranean diet, Dietary Approaches to Stop Hypertension (DASH), vegetarian dietary patterns, and the Nordic diet, have all been shown to have beneficial cardiometabolic outcomes [10, 11]. Key elements of the Nordic diet include whole grain cereals (especially rye, oats and barley), berries, fruit and vegetables, legumes, fish and shellfish, nuts, rapeseed oil, and low-fat dairy products [12, 13], which also align with the Nordic dietary guidelines.

The Nordic Nutrition Recommendations (NNR) summarize the available scientific evidence and provide regularly updated recommendations of nutritional intakes and physical activity [14–17]. The NNR aims to prevent non-communicable diseases, such as CVD, type 2 diabetes, obesity, and certain types of cancer. The NNR also emphasizes the importance of food patterns and nutrient intakes in combination with physical activity to reduce the risk of disease [18]. In addition, the NNR provides reference values and recommendations for total energy intake and intake of macronutrients, i.e. protein, carbohydrates, and fat expressed in energy percentage of total energy intake (excluding energy from alcohol), as well as vitamins and minerals, fiber, salt, alcohol and physical activity [17].

Previous studies have compared the food-based dietary guidelines from NNR, i.e. intakes on food group level, and a higher adherence to Nordic dietary patterns has been shown to reduce the total incidence of CVD, coronary heart disease, and stroke [12]. Herein we aimed to investigate the association between adherence to the daily recommended intake on nutrient level in NNR_1996_, NNR_2004_, NNR_2012_, and NNR_2023_ and the risk of MI and stroke in both women and men in a large population-based cohort.

## Material and methods

### Study design and study population

The Swedish National March Cohort (SNMC) was initiated in September 1997 as a four-day fundraising event by the Swedish Cancer Society. The cohort consists of 43,865 women and men who responded to an extensive 36-page questionnaire with information about lifestyle factors and medical history, which were collected in almost 3600 cities and villages around Sweden. The study design has been described previously [19].

### Inclusion and exclusion criteria

A total of 43,865 individuals gave informed consent and answered the questionnaire. We excluded individuals with an incorrect national registration number (*n* = 11), those younger than 18 years of age at enrollment (*n* = 1740), and those who had died (*n* = 8) or emigrated (*n* = 43) before the start of follow-up. This resulted in 42,063 eligible individuals. We further excluded individuals with a history of cardiovascular disease before the beginning of follow-up (heart failure, stroke, MI or CVD in general) (*n* = 4135) according to the International Classification of Diseases (ICD) versions 7 to 10 by linkage to the National Inpatient and Outpatient Register (ICD-7: 400–468; ICD-8: 330–334; ICD-9: 390–459; ICD-10: 100–199). Individuals with a previous cancer diagnosis other than non-melanoma skin cancer (*n* = 2677) were further excluded through linkage to the National Cancer Register (ICD-7:191 ICD-10: C44) before the start of follow-up. Finally, we excluded participants with missing food frequency questionnaire (FFQ) data (*n* = 17) or extreme values of total energy intake (± 3SDs from the mean value of the log-transformed energy intake) (*n* = 366) to ensure data quality. The final study sample for analyses included 34,898 individuals.

### Baseline characteristics

Baseline characteristics were assessed at enrollment and included sex (female/male), age (years), weight (kg), height (cm), level of education (≤ 13 years or > 13 years), and smoking status (never, former, current). Body mass index (BMI) was calculated as kg/m^2^. Self-reported information on prevalent hypertension, diabetes, and lipid disturbance (yes/no) were also defined in the questionnaire as having ever been treated by a physician for these conditions. Total physical activity was calculated as total metabolic energy turnover (MET) hours per day for time spending from sleeping to more vigorous activities summed up to 24 h. Each MET value for each intensity level from 0.9 MET for the lowest activity level up to 8 for the highest activity level was used to calculate total MET-hour/day [20].

### Adherence to the Nordic Nutrition Recommendations

Diet was assessed at baseline using a validated 85-item semi-quantitative FFQ [21]. Dietary supplements were not included. Participants were asked to report how often, on average, they consumed each item, by answering the question “How much on average do you eat/drink the following items”, with no specific time frame. Standard portion sizes were specified, and participants indicated frequency of consumption depending on the food item, from 0 to 7 or more servings per day, or as: 0, 1–3 times per month; 1–2, 3–4, 5–6 times per week or 1, 2, 3 or more times per day. The consumption of foods and beverages was then converted into nutritional data for daily intakes in grams or micrograms, including total energy, macro- and micronutrients, based on data from the Swedish Food Agency database (1998 version) [22]. Nutritional intakes were calculated once, and the same nutrient intakes were applied to calculate adherence to the four different NNR-scores, i.e. NNR_1996_, NNR_2004_, NNR_2012_, and NNR_2023_.

We estimated adherence to the NNR by creating a score based on 27 different nutrients and physical activity. In addition to a primary score based on the most recent NNR published in 2023 (NNR_2023_) [17], we also calculated NNR scores based on three previous versions, i.e., NNR_2012_ [16], NNR_2004_ [15], and NNR_1996_ [14]. The earliest version from 1996 (NNR_1996_) did not include physical activity. Our score with nine weighted groups (0–9 points), is based on the NNR–score that was originally developed by Fondell et al. [23], and later modified by Möller et al. [24]. The nine weighted groups were fat (including total fat, saturated fat, monounsaturated fat, polyunsaturated fat); protein; carbohydrates (including carbohydrates and sugar); vitamins (including 10 different vitamins); minerals (including 7 different minerals); fiber; salt; alcohol; and physical activity.

In the calculation of the NNR–scores we included total fat, i.e., saturated, mono-unsaturated and poly-unsaturated fat, carbohydrates, protein, and alcohol in energy percentage and daily intake of fiber and sodium, ten vitamins such as vitamin A, thiamine (vitamin B1), riboflavin (vitamin B2), niacin (vitamin B3), vitamin B6, folate, vitamin B12, vitamin C, vitamin D, and vitamin E, and seven minerals such as calcium, phosphorus, magnesium, potassium, iron, zinc, and selenium. All micronutrients were energy–adjusted on the log scale using the residual method for women and men separately [25], in order to remove extraneous variation by energy intake. The recommended intakes for all components included in the four different NNR–scores are shown in the Supplementary material, Table S1.

**Fig. 1:**
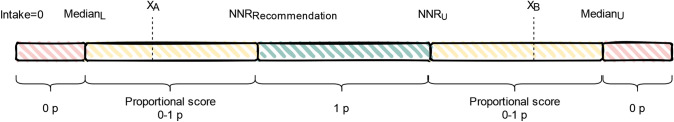
An overview of the calculation of the Nordic Nutrition Recommendations (NNR) score for each nutritional component and physical activity. The daily recommended intake (NNR_Recommendation_) and NNR upper (NNR_U_) represent the range of the recommended intake and upper cut-off points defined in NNR. Intake between these levels gives one point (perfect adherence). Median lower (Median_L_) and Median upper (Median_U_) define the median within the lowest and largest percentiles in our study population i.e., the extreme cut-off points for lower and upper intakes, respectively. Intakes outside the median cut-offs points give zero points (non-adherence). A proportional score (0–1) was calculated for intakes between the NNR_RI_ and the median cut-off (intermediate adherence) according to Eqs. 1 and 2. X_A_ and X_B_ corresponds to actual intakes within the range of the proportional score. Some of the nutrients have no defined upper limit. This figure is inspired by Möller et al. [23]

One point was given for perfect adherence, i.e., when the intake was within the daily recommended intake (RI) cut-off and the NNR upper (NNR_U_) level, see Fig. 1. For physical activity, adherence was defined as fulfilling the recommendation of moderate-to-vigorous physical activity of ≥ 150 min/week. Zero points were given for non-adherence, i.e. if the intake was above or below the median lower (Median_L_) or median upper (Median_U_) for the lowest and highest median percentiles for each nutrient and physical activity. A proportional score between 0 and 1 point was also calculated for intermediate adherence, i.e. intakes between the recommended intake and the median cut-offs according to Eqs. 1 and 2.

For intakes (or physical activity) below the recommendation, the score varied between 0 and 1:

1 $${\text{Proportional score }} = \left( {{\text{X}}_{{\text{A}}} - {\text{Median}}_{{\text{L}}} } \right)/\left( {{\text{NNR}}_{{{\text{Recommendation}}}} - {\text{ Median}}_{{\text{L}}} } \right)$$

For intakes above the NNR_U_, the score varied between 0 and 1:

2 $${\text{Proportional score }} = 1 - \left[ {\left( {{\text{X}}_{{\text{B}}} } \right. - \left. {{\text{NNR}}_{{\text{U}}} } \right)/\left( {{\text{Median}}_{{\text{U}}} } \right. - \left. {{\text{NNR}}_{{\text{U}}} } \right)} \right]$$

X_A_ and X_B_ correspond to actual intakes within the range of the proportional score. Vitamin C, folate, vitamin B12, vitamin B1, vitamin B2, magnesium, potassium, zinc, and fiber had no NNR_U_ defined in the NNR (Median_L_ was only used for the proportional score), while saturated fat, sugar, alcohol and sodium only had an upper limit, i.e., reverse recommendation (Median_U_ was only used for the proportional score).

Score components that included several individual recommendations (vitamins, minerals, fat, and carbohydrates) were summed and divided by the number of included recommendations to get equal weights for the nine variables in the final score. The points of the main nine variables were then summed into a total adherence NNR–score ranging between 0 and 9 points (0–8 points NNR_1996_). For the primary score NNR_2023_, low adherence was defined as < 6.3 points, moderate adherence as 6.3–7.2 points, and high adherence as > 7.2 points, to achieve a sufficient number of individuals in each group (corresponding to the 25% and 75% quartiles for the low and high and 50% for moderate) and to avoid to narrow intervals.

### Outcome measures and follow-up

The main outcome was the first incident MI or stroke (defined as a first hospitalization) occurring between October 1, 1997 and December 31, 2016. Participants were censored in case of emigration or death during the follow-up period. The national registration numbers enabled the cohort to be linked to the Swedish National Inpatient and Outpatient Register to determine non-fatal events, Swedish Cause of Death Register for fatal events, and Swedish Population Register for information on emigration. The following ICD codes; 410 (ICD-9), and I21 (ICD-10) were used to identify incident cases of MI, and 430, 433, 434 and 436 (ICD-9) and 160, 161, 163.0–163–5, 163.8–163.9 and 164 (ICD-10) for stroke, in the National Inpatient and Outpatient Register and the Cause of Death Register.

### Statistical analysis

Baseline characteristics are reported as means (standard deviation) for continuous variables, given that all were normally distributed based in visual inspection, or as frequencies and proportions for categorical variables. We fitted Cox proportional hazards regression models to estimate hazard ratios (HRs) with the corresponding 95% confidence intervals (CI) for associations between adherence to the NNR and incident MI and stroke during follow-up. Adherence to the NNR was included in the models as both a continuous variable and as categorical, respectively, with low adherence as the reference for the latter. Ties in survival times were handled using the Breslow method and the proportional hazards assumption was assessed by using Schoenfeld residuals for all covariates. If the assumption was violated, we included a stratification term for the corresponding variable in the model. Indeed, we observed a deviance from this assumption with respect to sex in the MI model and the categorical exposure itself for the stroke model, and thus also performed stratified analysis.

We ran multivariable analysis for controlling for potential confounders based on known risk factors for stroke and MI, including age, sex (not included in models stratified by sex), BMI, smoking status, education level, physical activity level in total METh/day, total daily energy intake, as well as self-reported history of diabetes, lipid disturbance and hypertension. We created a new categorical variable based on the median of each of NNR-score group and fitted it as a continuous variable in the model to examine linear trends across the groups. We further conducted stratified analyses by sex and investigated potential interactions between the exposure and sex on the multiplicative scale using the likelihood ratio test comparing nested models. We performed a sensitivity analysis to reduce the risk of possible reverse causation by excluding cases of MI or stroke that occurred during the first two years of follow-up. The statistical analyses were performed with Stata version 17.0 (Stata Corporation, Collage Station, TX, USA).

### Ethical approval

This study was approved by the Regional Ethical Review Board at Karolinska Institutet, Stockholm, Sweden (Dnr: 97-205 and 2017/796-31). Informed consent was obtained from all individual participants included in this study.

## Results

Demographic characteristics at baseline among all participants and by category of adherence to the NNR_2023_ are shown in Table 1. Of the 34,898 participants included in our analyses, 22,981 (66%) were female. The mean age was 49.4 (SD 15.8) years and the mean BMI was 24.5 kg/m^2^ (SD 3.5). Participants in the higher adherence groups were more likely to be younger, better educated and have a lower BMI, and less likely to be smokers compared to participants in the low adherence group (*p* < 0.001). Among women, 30.7% were classified as having high adherence and 16.6% as having low adherence, while the opposite distribution was seen among men, where 8.4% had high and 44.0% low adherence.

**Table 1:** Baseline characteristics of study participants in the Swedish National March Cohort by adherence to the Nordic Nutrition Recommendations 2023 (NNR_2023_) score

Characteristics	Total sample		Adherence to the NNR_2023_						*p*-value*
	*n* = 34,898		Low (< 6.3 points) (*n* = 9055)		Moderate (6.3–7.2 points) (*n* = 17,789)		High (> 7.2 points) (*n* = 8054)		
	Mean	(SD)	Mean	(SD)	Mean	(SD)	Mean	(SD)	
Age (years)	49.4	(15.8)	51.4	(15.7)	49.5	(16.0)	47.0	(15.1)	< 0.001
Physical activity (METh/day)	40.1	(13.8)	38.8	(14.4)	39.7	13.6	42.3	(13.3)	< 0.001
Alcohol (grams/month)	347	(626)	525	(1039)	315	(406)	220	(258)	< 0.001
Energy intake (kcal/day)	2042	(536)	2023	(569)	2052	(533)	2044	(503)	< 0.001

	*n*	(%)	*n*	(%)	*n*	(%)	*n*	(%)	
Sex									
Female	22,981	(65.9)	3815	(42.2)	12,115	(68.1)	7051	(87.6)	< 0.001
Male	11,917	(34.2)	5240	(57.9)	5674	(31.9)	1003	(12.5)	
BMI categories									
Normal weight, < 25 kg/m^2^	20,579	(61.6)	4538	(52.9)	10,494	(61.6)	5547	(71.3)	< 0.001
Overweight, ≥ 25 kg/m^2^	10,537	(31.6)	3286	(38.3)	5368	(31.5)	1883	(24.2)	
Obesity, ≥ 30 kg/m^2^	2271	(6.8)	758	(8.8)	1166	(6.9)	347	(4.5)	
Level of education, > 13 years	10,248	(29.7)	2243	(25.0)	5174	(29.4)	2831	(35.5)	< 0.001
Smoking status									
Never	20,914	(64.9)	4499	(55.7)	11189	(67.7)	5226	(68.8)	< 0.001
Former	8686	(27.0)	2599	(32.2)	4168	(25.2)	1919	(25.3)	
Current	2603	(8.1)	978	(12.1)	1175	(7.1)	450	(5.9)	
Risk factors									
Hypertension (yes)	3325	(9.8)	976	(11.3)	1778	(10.3)	571	(7.3)	< 0.001
Diabetes type II, (yes)	547	(1.7)	186	(2.2)	274	(1.6)	87	(1.1)	< 0.001
Lipid disturbance, (yes)	768	(2.3)	236	(2.8)	404	(2.4)	128	(1.6)	< 0.001

*MET* Metabolic equivalent of task; *BMI* body mass index

*Difference between adherence groups, *p*-values were obtained by analysis of covariance (ANOVA) for continuous variables, and by Chi^2^ test for categorical variables. Statistical significance is defined as *p* < 0.05

The proportion of missing values of the covariates was 7.7% for smoking, 4.9% for diabetes type II, 4.3% for BMI, 4.2% for lipid disturbance, 3.2% for hypertension, 1.0% for education, 0.28% for physical activity, and 0.14% for alcohol intake.

Table 2 presents the mean intakes and percentage of participants who adhered to each recommendation of the NNR_2023_. The mean score was 6.7 points among all, 6.9 points among women and 6.4 points among men. Mean intakes of saturated fat, monounsaturated fat, polyunsaturated fat, niacin, folate, vitamin D, vitamin E, potassium, selenium, and fiber were below the recommended levels, i.e., resulting in lower adherence to these recommendations. Almost all participants (> 80%) were within the recommendation for protein, vitamin A, thiamine, riboflavin, vitamin B6, vitamin B12, phosphorus, and sugar. The mean weekly physical activity was 100.1 min, where 29.1% reported completing the recommended ≥ 150 min per week. Somewhat higher mean scores of adherences were seen for NNR_1996_, NNR_2004_ and NNR_2012_ (Supplementary Table S1).

**Table 2:** Nordic Nutrition Recommendations 2023 (NNR_2023_) score; recommendations levels and cut-off points for adherence; energy adjusted mean intakes and percentage of adherence in the Swedish National March Cohort

NNR-score components	Cut-offs and adherence scores		Mean intake and adherence							*p*-value*
			All (*n* = 34,898)		Women (*n* = 22,981)		Men (*n* = 11,917)		Within NNR_2023_ (all)	
	Recommended intake (NNR_2023_)	Total score	Mean	(SD)	Mean	(SD)	Mean	(SD)	%	
Fat										
Total fat (E%)	25–40	0–1	25.5	(4.0)	25.5	(4.1)	25.5	(3.9)	55.8	0.42
Saturated fat (E%)	≤ 10		11.4	(2.3)	11.4	(2.4)	11.5	(2.3)	27.0	0.002
Monounsaturated fat (E%)	10–20		8.5	(1.5)	8.5	(1.5)	8.5	(1.5)	15.2	0.63
Polyunsaturated fat (E%)	5–10		3.3	(0.6)	3.3	(0.6)	3.3	(0.6)	1.1	0.09
Protein										
Protein (E%)	10–20	0–1	16.0	(1.9)	16.2	(1.9)	15.7	(1.9)	97.2	< 0.001
Carbohydrates										
Carbohydrates (E%)	45–60	0–1	58.8	(5.1)	58.9	(5.1)	58.5	(5.0)	60.4	< 0.001
Sugar (E%)	≤ 10		6.7	(2.9)	6.6	(2.7)	6.9	(3.2)	87.3	< 0.001
Vitamins										
Vitamin A (µg/d)		0–1	1286	(598)	1294	(600)	1272	(593)	83.3	0.001
Women	700–3000									
Men	800–3000									
Vitamin B_1_/Thiamine (mg/d)			1.6	(0.2)	1.6	(0.2)	1.8	(0.2)	99.9	< 0.001
Women	≥ 0.9									
Men	≥ 1.1									
Vitamin B_2_/Riboflavin (mg)			2.3	(0.6)	2.2	(0.5)	2.5	(0.6)	92.0	< 0.001
Women	≥ 1.6									
Men	≥ 1.6									
Vitamin B_3_/Niacin (mg/d)			14.5	(2.3)	13.9	(2.1)	15.6	(2.2)	35.3	< 0.001
Women	≥ 14									
Men	≥ 18									
Vitamin B_6_ (mg/d)			2.1	(0.3)	2.0	(0.3)	2.1	(0.3)	91.9	< 0.001
Women	1.6–12.5									
Men	1.8–12.5									
Folate (µg/d)	≥ 330		234.7	(43.5)	229.6	(43.3)	244.5	(42.3)	2.4	< 0.001
Vitamin B_12_ (µg/d)	≥ 4^a^		6.8	(2.6)	6.5	(2.6)	7.3	(2.7)	90.5	< 0.001
Vitamin C (mg/d)			95.9	(42.2)	98.4	(42.5)	91.0	(41.2)	40.3	< 0.001
Women	95–1000									
Men	110–1000									
Vitamin D (µg/d)	10–100		3.8	(1.4)	3.7	(1.4)	4.0	(1.6)	0.3	< 0.001
Vitamin E (mg/d)			5.2	(1.1)	5.3	(1.1)	5.1	(1.1)	0.1	< 0.001
Women	10–300^a^									
Men	11–300^a^									
Minerals										
Calcium (Ca) (mg/d)	950–2500	0–1	1147	(322)	1100	(290)	1236	(358)	72.5	< 0.001
Phosphorus (P) (mg/d)	525–3000^a^		1511	(262)	1447	(229)	1635	(275)	100.0	< 0.001
Magnesium (Mg) (mg/d)			316.5	(38.2)	303.6	(32.5)	341.2	(36.1)	48.0	< 0.001
Women	≥ 300^a^									
Men	≥ 350^a^									
Potassium (K) (mg/d)	≥ 3500^a^		3107	(520)	3046	(505)	3225	(527)	20.6	< 0.001
Iron (Fe) (mg/d)			15.9	(2.9)	15.1	(2.5)	17.6	(2.9)	66.8	< 0.001
Women	15–60									
Men	9–60									
Zinc (Zn) (mg/d)			10.4	(1.3)	10.0	(1.1)	11.3	(1.3)	35.7	< 0.001
Women	≥ 10									
Men	≥ 13									
Selenium (µg/d)			28.3	(7.0)	27.8	(6.8)	29.3	(7.1)	0.1	< 0.001
Women	75–255^a^									
Men	90–255^a^									
Fiber intake (g/d)		0–1	21.0	(4.6)	20.6	(4.4)	21.7	(4.9)	10.2	< 0.001
Women	≥ 25									
Men	≥ 35									
Sodium (mg/d)	≤ 1500^a^	0–1	1999	(310)	1894	(264)	2201	(292)	4.4	< 0.001
Alcohol (E%)	No safe limit	0–1	1.4	(1.6)	1.1	(1.3)	1.9	(1.8)	10.5	< 0.001
Physical activity recommendation										
Moderate- and vigorous activity, ≥ 5 MET (min/week)	≥ 150	0–1	100.1	(113.5)	92.0	(103.5)	115.7	(129.4)	29.1	< 0.001
Total score (min–max)		0–9	6.7	(0.6)	6.9	(0.6)	6.4	(0.6)		< 0.001

*E%* Energy percentage of total energy

*Difference between women and men using two-tailed *t*-test. Statistical significance is defined as *p* < 0.05

^a^Adequate intake (AI) was used instead of recommended intake

Incidence rates and hazard ratios (HR) of MI and stroke by adherence to the NNR_2023_ are shown in Table 3. In total, 1649 incident cases of MI and 2071 incident cases of stroke were observed during a mean follow-up period of 17.9 years (SD 3.7). For the continuous NNR_2023_-score, a 14% (HR: 0.86; 95% CI 0.78–0.95) reduced hazard of MI was found by each 1-point increase in the score in the fully adjusted model. In the categorical analysis of the score, participants in the high adherence group had a 28% lower hazard of MI (HR: 0.72; 95% CI 0.59–0.87; *p* for trend = 0.01) compared to the low adherence group. No associations were seen between adherence to the NNR and stroke for the continuous NNR_2023_. A 12% lower hazard of stroke (HR: 0.88; 95% CI 0.78–0.99; *p* for trend = 0.31) was observed for the moderate adherence group compared to the low adherence group. The results remained similar in sensitivity analyses excluding cases of stroke and MI occurring during the first two years of follow-up (data not shown).

**Table 3:** Incidence rates and hazard ratios of myocardial infarction and stroke for the adherence to the Nordic Nutrition Recommendations 2023 (NNR_2023_)

Total sample	No. of cases	Person-years	Model^a^		Model^b^	
			HR	(95% CI)	HR	(95% CI)
Myocardial infarction						
NNR_2023_-score (range 0–9)	1649	623,206	0.84	(0.77 to 0.90)	0.86	(0.78 to 0.95)
NNR_2023_ categories						
Low (< 6.3)	599	157,172	1.00	(Reference)	1.00	(Reference)
Moderate (6.3– 7.2)	840	318,310	0.93	(0.83 to 1.04)	1.00	(0.88 to 1.13)
High (> 7.2)	210	147,725	0.69	(0.58 to 0.81)	0.72	(0.59 to 0.87)
*P* for linear trend			< 0.01		0.01	
Stroke						
NNR_2023_-score (range 0–9)	2071	621,798	0.91	(0.85 to 0.98)	0.94	(0.87 to 1.03)
NNR_2023_ categories						
Low (< 6.3)	691	156,814	1.00	(Reference)	1.00	(Reference)
Moderate (6.3–7.2)	1003	318,223	0.83	(0.75 to 0.92)	0.88	(0.78 to 0.99)
High (> 7.2)	377	146,760	0.89	(0.78 to 1.01)	0.95	(0.81 to 1.11)
*P* for linear trend			0.02		0.31	
Female						
Myocardial infarction						
NNR_2023_-score (range 0–9)	737	418,638	0.75	(0.67 to 0.84)	0.77	0.67 to 0.89)
NNR_2023_ categories						
Low (< 6.3)	158	68,693	1.00	(Reference)	1.00	(Reference)
Moderate (6.3–7.2)	436	219,821	0.78	(0.65 to 0.93)	0.79	(0.64 to 0.99)
High (> 7.2)	143	130,124	0.58	(0.46 to 0.72)	0.59	(0.45 to 0.78)
*P* for linear trend			< 0.01		< 0.01	
Stroke						
NNR_2023_-score (range 0–9)	1149	416,343	0.92	(0.83 to 1.01)	0.98	(0.87 to 1.10)
NNR_2023_ categories						
Low (< 6.3)	224	68,195	1.00	(Reference)	1.00	(Reference)
Moderate (6.3–7.2)	624	219,003	0.78	(0.67 to 0.91)	0.84	(0.70 to 1.01)
High (> 7.2)	301	129,145	0.86	(0.72 to 1.02)	0.96	(0.78 to 1.18)
*P* for linear trend			0.17		0.92	
Male						
Myocardial infarction						
NNR_2023_-score (range 0–9)	912	204,568	0.92	(0.83 to 1.02)	0.97	(0.85 to 1.10)
NNR_2023_ categories						
Low (< 6.3)	441	88,478	1.00	(Reference)	1.00	(Reference)
Moderate (6.3–7.2)	404	98,489	0.99	(0.87 to 1.14)	1.09	(0.93 to 1.28)
High (> 7.2)	67	17,600	0.84	(0.65 to 1.08)	0.86	(0.64 to 1.17)
*P* for linear trend			0.33		1.00	
Stroke						
NNR_2023_-score (range 0–9)	922	205,455	0.91	(0.82 to 1.02)	0.91	(0.81 to 1.04)
NNR_2023_ categories						
Low (< 6.3)	467	88,620	1.00	(Reference)	1.00	(Reference)
Moderate (6.3–7.2)	379	99,220	0.87	(0.76 to 0.996)	0.90	(0.77 to 1.06)
High (> 7.2)	76	17,616	0.89	(0.70 to 1.14)	0.90	(0.68 to 1.18)
*P* for linear trend			0.07		0.21	

*HR* Hazards ratio; *CI* confidence interval, *PY* Person-years

^a^Adjusted for age (years) and sex (female/male, not in stratified analysis) at enrollment

^b^Adjusted for age (years), sex (female/male, not in stratified analysis), body mass index (kg/m^2^), level of education (≤ 13 years, > 13 years), smoking status (never, former, current), physical activity (METh/day), total daily energy intake (kcal/day), hypertension (yes/no), diabetes (yes/no), and lipid disturbance (yes/no)

In analyses stratified by sex, each 1-point increase in the NNR_2023_-score was associated with a 23% decreased hazard of MI among women (HR: 0.77; 95% CI 0.67–0.89) in fully adjusted models. Women with high adherence had a 41% (HR: 0.59; 95% CI 0.45–0.78; *p* for trend < 0.01) and a 21% lower hazard of MI (HR: 0.79; 95% CI 0.64–0.99; *p* for trend < 0.01), respectively, compared to women with low adherence. We found no associations between adherence to the NNR_2023_ and stroke among women, or between adherence to the NNR_2023_ and MI or stroke among men. The interaction analysis confirmed these findings and we observed significant interactions between the exposure and sex with regards for MI (*p*-value LR test = 0.03) in the multivariable analysis. No interaction was found for stroke (*p*-value LR test = 0.72). Results remained similar in sensitivity analyses excluding cases of stroke and MI occurring during the first two years of follow-up (data not shown).

The results remained overall similar for NNR_2012_ and NNR_2004,_ which are presented in Supplementary Tables S2 and S3. However, when we compared, the NNR_2012_ high adherence group to the low adherence group, a 25% lower hazard of MI (HR: 0.75; 95% CI 0.60–0.94), was found also among men. A similar association was seen in men for NNR_2004_ (HR: 0.78; 95% CI 0.63–0.97). In NNR_2004_, statistically significant associations were also found between adherence and risk of stroke. Among all, for each 1-point increase in the score, the risk of stroke decreased by 8% (HR: 0.92; 95% CI 0.85–1.00). Moderate and high adherence were associated with a lower risk of stroke compared to low adherence (HR: 0.83; 95% CI 0.73–0.93, and HR: 0.85; 95% CI 0.73–0.98, respectively). For NNR_1996_, Supplementary Table S4, no associations were seen among all participants, but the results were still statistically significant for MI among women.

## Discussion

In this large prospective cohort study, higher adherence to the NNR was associated with lower incidence of MI among all participants, and particularly among women. Our results also indicated that higher adherence was associated with lower incidence of stroke, although associations differed to some extend between editions of the NNR.

Adherence to different editions of the NNR resulted in similar associations with MI in our data. However, adherence to the NNR_1996_ was not significantly associated with MI among all participants, although the association was still significant in women. An explanation for this could be that several of the recommendations in NNR_1996_ were lower than in the more recent editions and physical activity was not included, leading to a different classification of participants. Associations between adherence to the NNR and stroke in our data differ somewhat depending on edition. However, point estimates were similar across versions of the NNR, although not statistically significant for all versions. The association between adherence to the NNR and stroke warrants further investigation.

When further comparing the recommendations across NNR editions, for seven nutrients, adequate intake (AI) is used instead of RI in the latest edition of NNR_2023_. AI is used when evidence is insufficient to establish an RI and can be used in line with RI values. However, it has a slightly greater uncertainty than RI [17]. Improved methodologies and updates of the scientific evidence [17] have led to at least 20% increased average requirements for nine nutrients between NNR_2012_ to the latest version. Additional key differences include a lower recommendation of energy coming from total fat in 1996 compared to the later editions i.e. 90.7% achieved the recommendation in 1996 compared to 55.8% in 2023. Recommendations for the other macronutrients have remained stable. The recommended levels of intake of micronutrients, including vitamin C, vitamin D and fiber, have increased in the later years. Sex-specific recommendations for fiber and vitamin C were added in NNR_2012_ and NNR_2023,_ respectively, resulting in more participants meeting the recommendation for fiber and vitamin C in NNR_1996_. The same trend was seen for vitamin D. The recommendation for sodium has been revised continuously, with the most recent being the lowest ever, resulting in only 4.4% of participants meeting the NNR_2023_ recommended intake. The recommendation for alcohol has been stable (< 5E%) from NNR_1996_ until the current version where no safe limit was introduced, leading to only 4.4% of participants meeting the recommendation compared to almost all previously. Finally, recommendations for physical activity were first introduced in NNR_2004_.

In the 2023 version of NNR, the authors recognized specific foods and nutrients associated with cardioprotective effects, that also indicative of the Nordic diet [17]. For example, whole grains from rye, oats and barley, fruits, vegetables and berries, dietary fiber, fish and seafood, nuts, rapeseed oil and low-fat dairy have shown beneficial effects on cardiovascular disease. Replacing animal-based intakes of fats (saturated fat) with plant-based fats (unsaturated fat) has also been shown to have a protective effect, and rapeseed oil has been associated with lower LDL-cholesterol compared with fats rich in saturated fat in randomized controlled trials. Furthermore, biomarker metabolites of fish, whole grains, and polyunsaturated fat have been associated with positive effects on serum lipids and blood glucose control [26]. Lastly, a high sodium intake increases the risk of hypertension, which is a risk factor for stroke and cardiovascular events [17].

The reasons for the sex differences found in the association between reported adherence to the NNR and MI are unclear. One possible explanation may be due to a sex-dependent response to components of the diet, or there could be other biological differences. A review concluded that platelet hyperactivity involved in the pathophysiology of stroke and myocardial infarction may be sex-related [27]. The same study also showed that components of the diet, such as polyunsaturated fat and flavonoids, can affect platelet functions in a sex-dependent manner. Another reason could be that women have a better dietary quality compared to men. This is reflected in our study where notably more women than men were categorized into the high adherence group. A narrative review also reported that women had better diet quality compared to men, and that food preferences influenced by social norms and biological differences may play a role in cardiovascular disease risk [28].

In a randomized control trial among individuals with high cholesterol, a healthy Nordic diet was shown to have beneficial effect on cardiovascular risk factors, such as serum blood lipids, insulin sensitivity and blood pressure [29]. Higher compliance to the NNR may—through a reduction in cardiovascular risk factors lower the risk of MI and potentially stroke as indicated by our data. Similar to our results, a diet in line with the Swedish nutrition recommendations and dietary guidelines that included components of saturated fat, polyunsaturated fat, fiber, sucrose, fish and shellfish, and fruit and vegetables, was associated with lower risk of non-fatal or fatal myocardial infarction, ischemic stroke and death from ischemic heart disease in another Swedish prospective cohort, including both men and women [30]. However, another Swedish cohort study in women [31] found no association with a higher adherence to a healthy Nordic food index and total CVD, including ischemic heart disease and stroke combined. Further, while we analyzed adherence based on nutrient intake, the healthy Nordic food index investigated in the study was based on selected food categories (fish, cabbage, rye bread, oatmeal, apple and pears, and root vegetables) [31].

In two large Danish cohort studies with over 55,000 men and women aged 50–64 years, a healthy Nordic diet has also been associated with a lower rate of MI [32] and stroke [33]. A difference between their study and ours is that instead of using food groups and cut-off values of the sex-specific median for each food item as in the previous studies, we used predefined cut-off values for sex-specific, nutrient-based recommendations and energy distribution from macronutrients according to NNR. Thus, we take into consideration the optimal nutritional intake from the overall diet aimed at preventing non-communicable diseases. Another strength of our study is that we used a continuous proportional score to the distance from the recommendations, reducing the risk of misclassification bias of absolute cut-off values.

There are several similarities between the NNR and dietary guidelines in other countries. For instance, both the NNR, a healthy diet according the WHO and Dietary Guidelines for Americans (DGA), are evidence-based and promote a diet that meets the nutritional needs to reduce the risk of non-communicable diseases [3, 17, 34]. All three encourage increased intake of fruits and vegetables, whole grains, healthy fats, lean protein, and limited intakes of saturated fat, added sugar, sodium and alcohol. However, specific recommendations for vitamin D may be more important in Nordic countries due to the limited sun exposure during winter, which may lead to a higher risk of vitamin D deficiency, compared to other countries [17]. Although, the NNR also includes specific dietary guidelines and nutritional needs of the Nordic and Baltic populations [17], the overall recommendations are generally in line with each other and our findings can most likely be applied to other populations outside the Nordic region.

The large study population with both women and men, the prospective design, and the long follow-up period are strengths of our study. This allowed us to perform sex-stratified analyses. Follow-up through linkage to well-maintained nationwide registries likely makes any possible non-differential misclassification of the outcome negligible. However, our study also has several limitations. Information on dietary intake was self-reported and only assessed at baseline. The participants may have changed their dietary habits during the follow-up period. In our study, participants volunteered during a fundraising event for cancer research, potentially leading to healthy volunteer bias and reduced generalizability of our findings. However, dietary intake in our study population was similar to that estimated in a large Swedish national survey conducted in 2010–2011 [35]. Although the daily intake of folate was low with only 2.4% achieving the recommended intake, it was comparable to the average intake in the Swedish population. However, the intakes of vitamin D, vitamin E and selenium were lower in our study compared to the intakes in the Swedish national survey. The reason may be that we measured dietary intake with an FFQ and they used a dietary record, which might capture nutrients differently. It may also be due to differences in versions of the food database used to calculate nutrient intakes.

Moreover, absolute nutritional values derived from an FFQ should be interpreted with caution. Although the FFQ used in our study included 85 different items, responses are limited to those listed in the FFQ, and to the use of standard portions sizes. There is a risk of both under- and over-reporting due to recall bias as participants report their usual intake based on their memory [36]. Due to social desirability bias, foods that are generally considered healthier, such as fruit and vegetables, tend to be overreported, whereas foods that are considerably unhealthy, such as junk foods, tend to be underreported [37]. Women may be more affected by social desirability than men [38], which may explain sex differences in reported adherence. On the other hand, potential misclassification of the exposure due to self-report of dietary intake is most likely non-differential with respect to those with and without subsequent MI and stroke. As a result, it would bias the estimates of association toward the null, leading to under-estimation of any causal associations. It is a strength that we energy–adjusted the nutrients in the NNR-score using the residual method [25], which minimizes bias from the total energy intake. The findings from our study could be of interest for the implementations of future dietary interventions to improve dietary habits.

## Conclusions

In conclusion, our findings indicate that adherence to NNR may reduce the risk of MI. The inverse association was more pronounced in women than men and in later editions of the NNR. The association between adherence to NNR and stroke was less pronounced and needs to be investigated further. These results support the role of continuously updating dietary recommendations to further target and reduce the risk of MI.

### Supplementary Information

Below is the link to the electronic supplementary material.

Supplementary file1 (PDF 318 KB)

## Data Availability

Data availability requests for anonymized datasets used and/or analyzed during the current study should be addressed to the principal investigators of the Swedish National March Cohort (YTL, WY, and RB). Data will be made available by reasonable request.

## References

[1] Afshin A, Sur PJ, Fay KA (2019). Health effects of dietary risks in 195 countries, 1990–2017: a systematic analysis for the Global Burden of Disease Study 2017. Lancet.

[2] World Health Organization (2023). Cardiovascular diseases.

[3] World Health Organization (2020). Healthy diet.

[4] Aune D, Giovannucci E, Boffetta P (2017). Fruit and vegetable intake and the risk of cardiovascular disease, total cancer and all-cause mortality—a systematic review and dose-response meta-analysis of prospective studies. Int J Epidemiol.

[5] Aune D, Keum N, Giovannucci E (2016). Nut consumption and risk of cardiovascular disease, total cancer, all-cause and cause-specific mortality: a systematic review and dose-response meta-analysis of prospective studies. BMC Med.

[6] Threapleton DE, Greenwood DC, Evans CEL (2013). Dietary fiber intake and risk of cardiovascular disease: systematic review and meta-analysis. BMJ.

[7] Aune D, Keum N, Giovannucci E (2016). Whole grain consumption and risk of cardiovascular disease, cancer, and all cause and cause specific mortality: systematic review and dose-response meta-analysis of prospective studies. BMJ.

[8] Strazzullo P, D’Elia L, Kandala N-B, Cappuccio FP (2009). Salt intake, stroke, and cardiovascular disease: meta-analysis of prospective studies. BMJ.

[9] Bowen KJ, Sullivan VK, Kris-Etherton PM, Petersen KS (2018). Nutrition and cardiovascular disease—an update. Curr Atheroscler Rep.

[10] Martínez-González MA, Gea A, Ruiz-Canela M (2019). The Mediterranean diet and cardiovascular health: a critical review. Circ Res.

[11] Kahleova H, Salas-Salvadó J, Rahelić D (2019). Dietary patterns and cardiometabolic outcomes in diabetes: a summary of systematic reviews and meta-analyses. Nutrients.

[12] Massara P, Zurbau A, Glenn AJ (2022). Nordic dietary patterns and cardiometabolic outcomes: a systematic review and meta-analysis of prospective cohort studies and randomised controlled trials. Diabetologia.

[13] Åkesson A, Andersen LF, Kristjánsdóttir ÁG (2013). Health effects associated with foods characteristic of the Nordic diet: a systematic literature review. Food Nutr Res.

[14] Sandström B, Aro A, Becker W (1996). Nordic Nutrition Recommendations 1996.

[15] Nordic Council of Ministers (2006). Nordic Nutrition Recommendations 2004: Integrating nutrition and physical activity.

[16] Nordic Council of Ministers (2014) Nordic Nutrition Recommendations 2012. Integrating nutrition and physical activity. Nordic Council of Ministers, Copenhagen

[17] Blomhoff R, Andersen R, Arnesen EK (2023). Nordic Nutrition Recommendations 2023.

[18] Christensen JJ, Arnesen EK, Andersen R (2020). The Nordic Nutrition Recommendations 2022 – principles and methodologies. Food Nutr Res.

[19] Trolle Lagerros Y, Hantikainen E, Mariosa D (2016). Cohort profile: The Swedish National March Cohort. Int J Epidemiol.

[20] Ainsworth BE, Haskell WL, Herrmann SD (2011). 2011 compendium of physical activities: a second update of codes and MET values. Med Sci Sports Exerc.

[21] Messerer M, Johansson S-E, Wolk A (2004). The validity of questionnaire-based micronutrient intake estimates is increased by including dietary supplement use in Swedish men. J Nutr.

[22] The Swedish Food Agency [Livsmedelsverket] (1998). Food composition tables.

[23] Fondell E, Christensen SE, Bälter O, Bälter K (2011). Adherence to the Nordic Nutrition Recommendations as a measure of a healthy diet and upper respiratory tract infection. Public Health Nutr.

[24] Möller E, Galeone C, Adami H-O (2012). The Nordic Nutrition Recommendations and prostate cancer risk in the Cancer of the Prostate in Sweden (CAPS) study. Public Health Nutr.

[25] Willett WC, Howe GR, Kushi LH (1997). Adjustment for total energy intake in epidemiologic studies. Am J Clin Nutr.

[26] Gürdeniz G, Uusitupa M, Hermansen K (2022). Analysis of the SYSDIET Healthy Nordic diet randomized trial based on metabolic profiling reveal beneficial effects on glucose metabolism and blood lipids. Clin Nutr.

[27] Gasperi V, Catani MV, Savini I (2020). Platelet responses in cardiovascular disease: sex-related differences in nutritional and pharmacological interventions. Cardiovasc Ther.

[28] Tindall AM, Stallings VA (2021). Sex differences in cardiovascular risk may be related to sex differences in diet patterns: a narrative review. Ann Hum Biol.

[29] Adamsson V, Reumark A, Fredriksson I-B (2011). Effects of a healthy Nordic diet on cardiovascular risk factors in hypercholesterolaemic subjects: a randomized controlled trial (NORDIET): diet and cardiovascular risk factors. J Intern Med.

[30] Hlebowicz J, Drake I, Gullberg B (2013). A high diet quality is associated with lower incidence of cardiovascular events in the Malmö Diet and Cancer Cohort. PLoS ONE.

[31] Roswall N, Sandin S, Scragg R (2015). No association between adherence to the healthy Nordic food index and cardiovascular disease amongst Swedish women: a cohort study. J Intern Med.

[32] Gunge VB, Andersen I, Kyrø C (2017). Adherence to a healthy Nordic food index and risk of myocardial infarction in middle-aged Danes: the diet, cancer and health cohort study. Eur J Clin Nutr.

[33] Hansen CP, Overvad K, Kyrø C (2017). Adherence to a healthy Nordic diet and risk of stroke: a Danish cohort study. Stroke.

[34] Department of Agriculture and US Department of Health and Human Services. Dietary Guidelines for Americans, 2020–2025, 9th Edition. December 2020

[35] Amcoff E, Edberg A, Enghardt Barbieri H et al (2012) Riksmaten - vuxna 2010–11 Livsmedels- och näringsintag bland vuxna i Sverige. Livsmedelsverket, Uppsala

[36] Naska A, Lagiou A, Lagiou P (2017). Dietary assessment methods in epidemiological research: current state of the art and future prospects. F1000Research.

[37] Warnecke RB, Johnson TP, Chávez N (1997). Improving question wording in surveys of culturally diverse populations. Ann Epidemiol.

[38] Tang JS, Haslam RL, Ashton LM (2022). Gender differences in social desirability and approval biases, and associations with diet quality in young adults. Appetite.

