# Supplementary material for: Nordic Nutrition Recommendations and risk of myocardial infarction and stroke: a prospective cohort study
Source: Eur J Nutr. 2024 Feb 14;63(4):1151–62. doi: 10.1007/s00394-024-03337-7 (PMC11139687; doi:10.1007/s00394-024-03337-7)
Supplement: Supplementary file 1 — Supplementary file1 (PDF 318 KB) [file 394_2024_3337_MOESM1_ESM.pdf]

Supplementary material

Supplementary Table S1. Differences in Nordic Nutrition Recommendations (NNR) from 1996, 2004, 2012 and 2023.

NNR score components	Recommended intake cut-offs and percentage within the NNR							
	**NNR_{1996}**	Within the NNR_{1996} (%)	**NNR_{2004}**	Within the NNR_{2004} (%)	**NNR_{2012}**	Within the NNR_{2012} (%)	**NNR_{2023}**	Within the NNR_{2023} (%)
**Fat**								
Total fat (E%)	20-30	90.7	25-35	54.9	25-40	55.8	25-40	55.8
Saturated fat (E%)	≤10	27.0	≤10	27.0	≤10	27.0	≤10	27.0
Monounsaturated fat (E%)	10-15	15.2	10-15	15.2	10-20	15.2	10-20	15.2
Polyunsaturated fat (E%)	5-10	1.1	5-10	1.1	5-10	1.1	5-10	1.1
**Protein**								
Protein (E%)	10-15	97.2	10-20	97.2	10-20	97.2	10-20	97.2
**Carbohydrates**								
Carbohydrates (E%)	55-60	57.2	50-60	57.2	45-60	60.4	45-60	60.4
Sugar (E%)	≤10	87.3	≤10	87.3	≤10	87.3	≤10	87.3
**Vitamins**								
Vitamin A (µg/d)		74.8		80.2		80.2		83.3
Women	800-3000		700-3000		700–3000		700–3000	
Men	900-3000		900-3000		900–3000		800–3000	
Vitamin B_1/Thiamine (mg/d)		98.0		98.0		98.0		99.9
Women	≥1.1		≥1.1		≥1.1		≥0.9	
Men	≥1.4		≥1.4		≥1.4		≥1.1	
Vitamin B_2/Riboflavin (mg)		96.5		95.7		95.7		92.0
Women	≥1.3		≥1.3		≥1.3		≥1.6	
Men	≥1.6		≥1.7		≥1.7		≥1.6	
Vitamin B_3/Niacin (mg/d)		22.9		20.4		22.9		35.3
Women	≥15		15-30		15-35		≥14	
Men	≥18		19-30		18-35		≥18	
Vitamin B_6 (mg/d)		99.7		99.7		99.7		91.9

Women	1.2-50		1.2-25		1.2-25		1.6-12.5	
Men	1.5-50		1.6-25		1.5-25		1.8-12-5	
Folate (µg/d)	≥300	6.6	≥300	6.6	≥300	6.6	≥330	2.4
Vitamin B_{12} (µg/d)	≥2.0	99.6	≥2.0	99.5	≥2.0	99.5	≥4.0[a]	90.5
Vitamin C (mg/d)		80.7		65.8		65.8		40.3
Women	≥60		≥75		≥75		95-1000	
Men	≥60		≥75		≥75		110-1000	
Vitamin D (µg/d)	≥5	17.2	10-50	0.3	10–100	0.3	10-100	0.3
Vitamin E (mg/d)		1.3		1.3		1.3		0.1
Women	≥8		8-300		8-300		10-300[a]	
Men	≥10		10-300		10-300		11-300[a]	
**Minerals**								
Calcium (Ca) (mg/d)	800-2500	88.5	800-2500	88.5	800-2500	88.5	950-2500	72.5
Phosphorus (P) (mg/d)	≥600	100.0	600-4000	100.0	600-3000	100.0	525-3000[a]	100.0
Magnesium (Mg) (mg/d)		64.1		64.1		64.1		48.0
Women	≥280		≥280		≥280		≥300[a]	
Men	≥350		≥350		≥350		≥350[a]	
Potassium (K) (mg/d)		37.7		37.7		37.7		20.6
Women	≥3100		≥3100		≥3100		≥3500	
Men	≥3500		≥3500		≥3500		≥3500[a]	
Iron (Fe) (mg/d)		99.2		99.6		99.6		66.8
Women	10-60		9-25		9–60		15-60	
Men	10-60		9-25		9–60		9-60	
Zinc (Zn) (mg/d)		98.4		98.4		98.4		35.7
Women	≥7		≥7		≥7		≥10	
Men	≥9		≥9		≥9		≥13	
Selenium (µg/d)		3.3		3.3		0.6		0.1
Women	40-300		40-300		50-300		75-255[a]	
Men	50-300		50-300		60-300		90-255[a]	
**Fibre intake** (g/d)		56.2		17.8		10.2		10.2
Women	≥20		≥25		≥25		≥25	
Men	≥20		≥25		≥35		≥35	
**Sodium** (mg/d)		51.8		90.8		90.8		4.4
Women	≤2000		≤2400		≤2400		≤1500[a]	

Men	≤2000		≤2800		≤2400		≤1500[a]	
**Alcohol** (E%)	<5	96.8	<5	96.8	<5	96.8	No safe limit	10.5
**Physical activity recommendation**								
Moderate and vigorous activity, ≥5 MET (min/week)	Not included		≥150	29.1	≥150	29.1	≥150	29.1
**Total score**	7.1[2]	(SD 0.5)	7.4[3]	(SD 0.6)	7.3[3]	(SD 0.6)	6.7[3]	(SD 0.6)

Abbreviations: E%, energy percentage of total energy.
[a]Adequate intake (AI) was used instead of recommended intake.
[1]Moderate activity was defined as activities corresponding to 5 MET (Metabolic equivalent task) and vigorous as activities >6 MET.
[2]Including nutrient variables only, range 0-8.
[3]Including nutrient variables and physical activity, range 0-9.

Supplementary Table S2. Incidence rates and hazard ratios of myocardial infarction and stroke for the adherence to the Nordic Nutrition Recommendations 2012 (NNR_{2012}).

	No. of cases	Person-years	Model[a]		Model[b]	
**Total sample**			HR	(95% CI)	HR	(95% CI)
Myocardial infarction						
NNR-score (range 0-9)	1,649	623,206	0.81	(0.75 to 0.88)	0.83	(0.76 to 0.92)
NNR categories						
Low (<6.9)	563	150,071	1.00	(reference)	1.00	(reference)
Moderate (6.9-7.7)	787	301,820	0.89	(0.80 to 0.99)	0.91	(0.80 to 1.04)
High (>7.7)	299	171,316	0.69	(0.60 to 0.85)	0.71	(0.60 to 0.84)
P for linear trend			<0.01		<0.01	
Stroke						
NNR-score (range 0-9)	2,071	621,798	0.90	(0.84 to 0.97)	0.92	(0.84 to 1.00)
NNR categories						
Low (<6.9)	622	150,034	1.00	(reference)	1.00	(reference)
Moderate (6.9-7.7)	987	301,161	0.90	(0.82 to 1.00)	0.91	(0.81 to 1.03)
High (>7.7)	462	170,602	0.88	(0.78 to 0.99)	0.89	(0.77 to 1.03)
P for linear trend			0.04		0.11	
**Female**						
Myocardial infarction						
NNR-score (range 0.9)	737	418,638	0.74	(0.66 to 0.84)	0.78	0.67 to 0.90)
NNR categories						
Low (<6.9)	176	75,860	1.00	(reference)	1.00	(reference)
Moderate (6.9-7.7)	405	209,645	0.76	(0.64 to 0.91)	0.76	(0.61 to 0.93)
High (>7.7)	156	133,133	0.61	(0.49 to 0.76)	0.65	(0.50 to 0.84)
P for linear trend			<0.01		<0.01	
Stroke						
NNR-score (range 0.9)	1,149	416,343	0.89	(0.80 to 0.98)	0.95	(0.84 to 1.07)
NNR categories						
Low (<6.9)	240	75,325	1.00	(reference)	1.00	(reference)
Moderate (6.9-7.7)	615	208,645	0.85	(0.73 to 0.98)	0.91	(0.76 to 1.09)
High (>7.7)	294	132,373	0.84	(0.71 to 1.00)	0.92	(0.75 to 1.13)

P for linear trend			0.07		0.46	
**Male**						
Myocardial infarction						
NNR-score (range 0-9)	912	204,568	0.87	(0.79 to 0.97)	0.90	(0.80 to 1.01)
NNR categories						
Low (<6.9)	387	74,211	1.00	(reference)	1.00	(reference)
Moderate (6.9-7.7)	382	92,174	0.96	(0.83 to 1.10)	1.00	(0.85 to 1.18)
High (>7.7)	143	38,183	0.76	(0.62 to 0.92)	0.75	(0.60 to 0.94)
P for linear trend			0.01		0.03	
Stroke						
NNR-score (range 0-9)	922	205,455	0.92	(0.83 to 1.02)	0.90	(0.80 to 1.02)
NNR categories						
Low (<6.9)	382	74,709	1.00	(reference)	1.00	(reference)
Moderate (6.9-7.7)	372	92,517	0.95	(0.82 to 1.10)	0.91	(0.77 to 1.07)
High (>7.7)	168	38,230	0.91	(0.76 to 1.10)	0.87	(0.71 to 1.08)
P for linear trend			0.30		0.17	

Abbreviations: HR, hazards ratio; CI, confidence interval, PY Person-years.

[a]Adjusted for age (years) and sex (female/male, not in stratified analysis) at enrollment.

[b]Adjusted for age (years), sex (female/male, not in stratified analysis), body mass index (kg/m^2), level of education (≤13 years, >13 years), smoking status (never, former, current), physical activity (METh/day), total daily energy intake (kcal/day), hypertension (yes/no), diabetes (yes/no), and lipid disturbance (yes/no).

Supplementary Table S3. Incidence rates and hazard ratios of myocardial infarction and stroke for the adherence to the Nordic Nutrition Recommendations 2004 (NNR_{2004}).

	No. of cases	Person-years	Model[a]		Model[b]	
**Total sample**			HR	(95% CI)	HR	(95% CI)
Myocardial infarction						
NNR-score (range 0-9)	1,649	623,206	0.83	(0.77 to 0.89)	0.84	(0.77 to 0.92)
NNR categories						
Low (<7.0)	442	142,674	1.00	(reference)	1.00	(reference)
Moderate (7.0-7.9)	900	332,461	0.89	(0.79 to 0.998)	0.89	(0.78 to 1.02)
High (>7.9)	307	148,072	0.70	(0.60 to 0.81)	0.72	(0.61 to 0.86)
P for linear trend			<0.01		<0.01	
Stroke						
NNR-score (range 0-9)	2,071	621,798	0.91	(0.85 to 0.97)	0.92	(0.85 to 0.999)
NNR categories						
Low (<7.0)	543	142,137	1.00	(reference)	1.00	(reference)
Moderate (7.0-7.9)	1,082	332,164	0.86	(0.77 to 0.95)	0.83	(0.73 to 0.93)
High (>7.9)	446	147,497	0.86	(0.76 to 0.97)	0.85	(0.73 to 0.98)
P for linear trend			0.01		0.03	
**Female**						
Myocardial infarction						
NNR-score (range 0.9)	737	418,638	0.76	(0.67 to 0.85)	0.79	(0.68 to 0.90)
NNR categories						
Low (<7.0)	207	95,236	1.00	(reference)	1.00	(reference)
Moderate (7.0-7.9)	417	228,306	0.84	(0.71 to 0.99)	0.82	(0.67 to 1.00)
High (>7.9)	113	95,096	0.65	(0.52 to 0.82)	0.69	(0.53 to 0.91)
P for linear trend			<0.01		0.01	
Stroke						
NNR-score (range 0.9)	1,149	416,343	0.89	(0.81 to 0.98)	0.95	(0.85 to 1.07)
NNR categories						
Low (<7.0)	313	94,435	1.00	(reference)	1.00	(reference)
Moderate (7.0-7.9)	616	227,423	0.81	(0.71 to 0.93)	0.82	(0.70 to 0.97)
High (>7.9)	220	94,484	0.84	(0.70 to 0.99)	0.86	(0.70 to 1.06)

P for linear trend			0.03		0.14	
**Male**						
Myocardial infarction						
NNR-score (range 0-9)	912	204,568	0.89	(0.80 to 0.98)	0.90	(0.80 to 1.01)
NNR categories						
Low (<7.0)	235	47,438	1.00	(reference)	1.00	(reference)
Moderate (7.0-7.9)	483	104,155	0.95	(0.81 to 1.10)	0.99	(0.83 to 1.18)
High (>7.9)	194	52,976	0.75	(0.62 to 0.91)	0.78	(0.63 to 0.97)
P for linear trend			<0.01		0.03	
Stroke						
NNR-score (range 0-9)	922	205,455	0.93	(0.84 to 1.02)	0.90	(0.80 to 1.01)
NNR categories						
Low (<7.0)	230	47,701	1.00	(reference)	1.00	(reference)
Moderate (7.0-7.9)	466	104,741	0.93	(0.79 to 1.08)	0.85	(0.71 to 1.01)
High (>7.9)	226	53,012	0.90	(0.75 to 1.08)	0.85	(0.69 to 1.05)
P for linear trend			0.27		0.15	

Abbreviations: HR, hazards ratio; CI, confidence interval, PY Person-years.

[a]Adjusted for age (years) and sex (female/male, not in stratified analysis) at enrollment.

[b]Adjusted for age (years), sex (female/male, not in stratified analysis), body mass index (kg/m^2), level of education (≤13 years, >13 years), smoking status (never, former, current), physical activity (METh/day), total daily energy intake (kcal/day), hypertension (yes/no), diabetes (yes/no), and lipid disturbance (yes/no).

Supplementary Table S4. Incidence rates and hazard ratios of myocardial infarction and stroke for the adherence to the Nordic Nutrition Recommendations 1996 (NNR_{1996}).

	No. of cases	Person-years	Model[a]		Model[b]	
**Total sample**			HR	(95% CI)	HR	(95% CI)
Myocardial infarction						
NNR-score (range 0-8)	1,649	623,206	0.87	(0.80 to 0.95)	0.91	(0.82 to 1.00)
NNR categories						
Low (<6.9)	421	155,725	1.00	(reference)	1.00	(reference)
Moderate (6.9-7.4)	860	313,619	0.86	(0.77 to 0.98)	0.91	(0.79 to 1.04)
High (>7.4)	368	153,862	0.83	(0.72 to 0.95)	0.85	(0.72 to 1.01)
P for linear trend			0.01		0.06	
Stroke						
NNR-score (range 0-8)	2,071	621,798	0.93	(0.86 to 1.01)	0.96	(0.87 to 1.05)
NNR categories						
Low (<6.9)	458	155,682	1.00	(reference)	1.00	(reference)
Moderate (6.9-7.4)	1,088	312,993	0.98	(0.87 to 1.09)	1.02	(0.90 to 1.16)
High (>7.4)	525	153,123	0.93	(0.82 to 1.06)	0.96	(0.82 to 1.11)
P for linear trend			0.31		0.62	
**Female**						
Myocardial infarction						
NNR-score (range 0-8)	737	418,638	0.82	(0.72 to 0.92)	0.85	(0.73 to 0.99)
NNR categories						
Low (<6.9)	153	87,964	1.00	(reference)	1.00	(reference)
Moderate (6.9-7.4)	336	200,855	0.74	(0.61 to 0.90)	0.80	(0.64 to 1.01)
High (>7.4)	248	129,819	0.73	(0.60 to 0.89)	0.75	(0.59 to 0.96)
P for linear trend			<0.01		0.02	
Stroke						
NNR-score (range 0-8)	1,149	416,343	0.86	(0.78 to 0.96)	0.93	(0.83 to 1.06)
NNR categories						
Low (<6.9)	210	87,572	1.00	(reference)	1.00	(reference)
Moderate (6.9-7.4)	546	199,801	0.89	(0.76 to 1.04)	0.99	(0.75 to 1.13)
High (>7.4)	393	128,969	0.85	(0.76 to 1.00)	0.92	(0.75 to 1.13)

P for linear trend			0.06		0.92	
**Male**						
Myocardial infarction						
NNR-score (range 0-8)	912	204,568	0.93	(0.83 to 1.05)	0.98	(0.85 to 1.13)
NNR categories						
Low (<6.9)	268	67,761	1.00	(reference)	1.00	(reference)
Moderate (6.9-7.4)	524	112,764	0.95	(0.82 to 1.11)	0.98	(0.83 to 1.16)
High (>7.4)	120	24,043	0.90	(0.72 to 1.11)	0.95	(0.74 to 1.21)
P for linear trend			0.33		0.67	
Stroke						
NNR-score (range 0-8)	922	205,455	1.02	(0.90 to 1.15)	0.99	(0.86 to 1.14)
NNR categories						
Low (<6.9)	248	68,110	1.00	(reference)	1.00	(reference)
Moderate (6.9-7.4)	542	113,191	1.06	(0.91 to 1.23)	1.05	(0.88 to 1.24)
High (>7.4)	132	24,154	1.05	(0.85 to 1.29)	1.00	(0.78 to 1.27)
P for linear trend			0.53		0.83	

Abbreviations: HR, hazards ratio; CI, confidence interval, PY Person-years.

[a]Adjusted for age (years) and sex (female/male, not in stratified analysis) at enrollment.

[b]Adjusted for age (years), sex (female/male, not in stratified analysis), body mass index (kg/m^2), level of education (≤13 years, >13 years), smoking status (never, former, current), physical activity (METh/day), total daily energy intake (kcal/day), hypertension (yes/no), diabetes (yes/no), and lipid disturbance (yes/no).
